# Isorhamnetin Induces Melanoma Cell Apoptosis via the PI3K/Akt and NF-*κ*B Pathways

**DOI:** 10.1155/2020/1057943

**Published:** 2020-05-05

**Authors:** Ran Duan, Xiao Liang, Bangda Chai, Yiwen Zhou, Hengyu Du, Yingjun Suo, Zhaohuan Chen, Qingfeng Li, Xiaolu Huang

**Affiliations:** Department of Plastic & Reconstructive Surgery, Shanghai Ninth People's Hospital, Shanghai Jiao Tong University School of Medicine, 639 Zhizaoju Road, Shanghai 200011, China

## Abstract

Malignant melanoma is characterized by its bad prognosis for aggressiveness, drug resistance, and early metastasis. Isorhamnetin (3′-methoxy-3,4′,5,7-tetrahydroxyflavone; IH) is a natural flavonoid that has been investigated for its antitumor effects in breast cancer, colon cancer, and gastric cancer through inducing cell apoptosis. Given its role in tumor inhibition, no research has been conducted concerning its effect against melanoma. In the present study, we found that IH could significantly inhibit B16F10 cell proliferation and migration and induce B16F10 cell apoptosis. The examination on molecular mechanism revealed that IH could suppress the phosphorylation of Akt and the translocation of NF-*κ*B, which are key factors in apoptosis-related pathways. We also detected that this process was related to the bifunctional 6-phosphofructo-2-kinase/fructose-2,6-bisphosphatases 4 (PFKFB4) by PFKFB4 knockdown experiment. In line with in vitro study, we further provided that IH effectively inhibited tumor growth in vivo. Taken together, IH was proven to induce melanoma cell apoptosis in vitro and in vivo, which may serve as a potential agent in malignant melanoma treatment in the future.

## 1. Introduction

Malignant melanoma is the most fatal form of skin cancer, which accounts for 80% death of all types of skin cancer and 3% death of all malignant tumors. According to the World Health Organization (WHO), the incidence of melanoma has been doubled every one to two decades. [1] Currently, there are different types of treatments for patients depending on the stage of melanoma. However, these treatments are limited due to tumor aggressiveness, tumor recurrence, and drug resistance. The 5-year survival rate of metastatic melanoma patients is less than 10%, with an overall median survival of 4-6 months. [2–4] Thus, novel treating strategies are urgently needed.

Considering that tumor development relies largely on irregular cell apoptosis, much attention has been paid to apoptotic mechanism. The phosphatidylinositol-3-OH kinase (PI3K) pathway is a well-acknowledged key signal pathway in cell cycle, apoptosis, and proliferation, which makes it an important target in multiple tumor types. It was reported that the activity of the PI3K/Akt pathway is increased in 70% sporadic melanoma. [1] The kinase Akt functions as a central integrator of PI3K signalling to modulate downstream effectors, which are primary regulators of protein synthesis and cell growth. [1]

Flavonoids are a group of chemicals containing flavone (2-Phenyl-4H-chromen-4-one) that have been used as Chinese traditional medicine. Isorhamnetin (3′-methoxy 3,4′,5,7-tetrahydroxyflavone; IH) is a kind of flavonoids that is separated and purified from the medicinal plants such as Persicaria thunbergii H. and Elaeagnus rhamnoides (L.) A. Nelson. [5] Elaeagnus rhamnoides (L.) A. Nelson has been reported to have anticancer effects; the probable mechanism includes oxidation action, antiproliferation action, and anti-inflammatory action. [6, 7] Isorhamnetin shows a variety of pharmacological activities such as cardiovascular protection, antioxidant, anticholinesterase, and antibacterial. [8, 9] Besides, its anticancer effect has been proven in esophageal cancer [10], colon cancer [11], breast cancer [12], and lung cancer [13]. However, to the best of our knowledge, there is no research focusing on the inhibitory effect of IH in melanoma, and the mechanism underlying has not yet been clarified.

In the present study, we investigated the effect of IH on B16F10 melanoma cell proliferation, apoptosis, and migration. The apoptosis-related proteins, such as Bax, Bcl-2, and Caspase-3, and the alterations of the PI3K/Akt and NF-*κ*B pathways in B16F10 cells after IH treatment were examined. We also discovered that such bioactivity of IH was related to bifunctional 6-phosphofructo-2-kinase/fructose-2,6-bisphosphatases 4 (PFKFB4). To further confirm the suppressive effect of IH in vivo, C57BL/6 mice were adopted for B16F10 cell injection. A remarkable difference in tumor size was observed 7 days after IH treatment, and a decrease of Ki67 immunofluorescence staining was detected in the IH-treated group, which indicated the inhibitive effect of IH on cell proliferation in vivo.

 

## 2. Materials and Methods

### 2.1. Reagents

Isorhamnetin was purchased from Shanghai Yuanye Biological Technology Co., Ltd. (Shanghai, China); samples containing 98% or higher isorhamnetin were used during the whole in vitro experiments. The compound was dissolved in dimethyl sulfoxide (DMSO; Sigma-Aldrich, St. Louis, MO, USA) and diluted to 10 mmol/L, stored at -20°C until needed. The final concentration of DMSO did not exceed 0.1% in experiments (this concentration was confirmed to have no cytotoxic effect). The controls were treated with the same amount of DMSO (0.1%) as used in corresponding experiments. A CCK-8 kit was purchased from Dojindo (Tokyo, Japan). 0.25% Trypsin-EDTA was purchased from Gibco (Grand Island, NY, USA). Insulin-like growth factor-1 (IGF-1) was obtained from PeproTech (Rocky Hill, New Jersey, USA). The primary antibodies were as follows: PI3K (Abcam, Cambridge, UK 1 : 1000), AkT (Abcam, Cambridge, UK 1 : 1000), phosphorylated (p) AkT (ser473) (Abcam, Cambridge, UK 1 : 1000), Bax (Abcam, Cambridge, UK 1 : 1000), Bcl-2 (Abcam, Cambridge, UK 1 : 2000), Caspase-3 (Abcam, Cambridge, UK 1 : 5000), NF-*κ*B (Abcam, Cambridge, UK 1 : 1000), and PFKFB4 (Abcam, Cambridge, UK 1 : 500).

### 2.2. Cell Lines and Culture

The B16F10 cell line was obtained from the Chinese Academy of Sciences (Beijing, China). B16F10 cells were cultured in Dulbecco's modified Eagle's medium (DMEM; HyClone, Thermo Fisher Scientific, Waltham, MA, USA) with 10% fetal bovine serum (FBS; HyClone, Thermo Fisher Scientific, Waltham, MA, USA), 100 U/mL penicillin, and 100 mg/L streptomycin (Gibco, Grand Island, NY, USA). Cells were incubated at 37°C in a humidified atmosphere with 5% CO_2_.

### 2.3. Cell Counting Kit-8 (CCK-8) Assay

5 × 10^3^ B16F10 cells/well in a 96-well plate were treated with various concentrations of IH (0, 10, 25, 50, and 100 *μ*mol/L) for 48 h. The control group was treated with DMSO. At the appropriate timepoints, cell proliferation assay was performed by the addition of 10 *μ*L CCK-8 solution to each well, followed by incubation at 37°C for 2 h. Absorbance at a wavelength of 450 nm was measured using a microplate reader (Synergy 2 Multi-Mode Microplate Reader; BioTek, Winooski, VT, USA).

### 2.4. Clonogenic Assay

Exponentially growing cells were harvested, counted to 1000 cells, and seeded into plates. Cells were allowed to grow at 37°C with 5% CO_2_ overnight. Then, cells were treated with different concentrations of IH (0-100 *μ*mol/L) for an additional 24 h. After incubation, the cells were rinsed with PBS and replaced by fresh medium containing 10% FBS. At the 7th day of incubation, cells were fixed with 3.7% paraformaldehyde and stained with the crystal violet solution. Relative survival was calculated from the number of single cells that formed colonies of >50 cells.

### 2.5. Western Blot Analysis

Cells were seeded onto 6-well plates following treatment with 100 *μ*mol/L IH or DMSO, and proteins were harvested and collected by radioimmunoprecipitation assay (RIPA) lysis buffer. The protein concentrations were determined by a Bicinchoninic Acid Protein Assay kit (Beyotime, P0010). Subsequently, 30 *μ*g of each protein sample was added to 5x sodium dodecyl sulfate polyacrylamide gel electrophoresis gel (SDS-PAGE). Following electrophoresis at 100 V for 2 h, the proteins were transferred to polyvinylidene difluoride (PVDF) membranes (Bedford, MA, USA) at 300 mA for 90 min. The membranes were then blocked and incubated with primary antibody at 4°C overnight, washed three times with TBST, incubated for 2 h at room temperature with secondary antibody, and washed again three times. Protein expression was detected with an enhanced chemiluminescence detection system (Tanon, Shanghai, China).

### 2.6. Extraction of Nuclear and Cytosolic Fractions

The extraction and isolation of nuclear and cytoplasmic proteins were performed according to the manufacturer's instructions using a nuclear and cytoplasmic protein extraction kit (Pierce, Thermo Fisher Scientific, Waltham, MA, USA). Briefly, after treatment, cells were harvested and washed twice with PBS, then collected by centrifugation at 500 × g for 3 min. Cell pellets were resuspended in 500 *μ*L cytoplasmic extraction reagent I, vortexed, and incubated for 10 min on ice. 27.5 *μ*L cytoplasmic extraction reagent II was then added, vortexed, and incubated for 1 min on ice. After centrifugation at 15 000 × g for 5 min at 4°C, the supernatants which contained the cytoplasmic extract were removed and stored at -80°C. The pellets containing nuclear fraction were resuspended in 250 *μ*L of nuclear extract reagent. After being vortexed for 15 seconds every 10 minutes for a total of 40 minutes, samples were centrifuged at 15 000 × g for 10 min at 4°C. The supernatants were removed and stored at -80°C until use. The validation of the method used to isolate the cytoplasmic and nuclear extracts (histone-H3 was used as a loading control for nuclear proteins, and *β*-actin was used as the loading control for cytoplasm proteins) was checked using Western blot analysis.

### 2.7. Flow Cytometry Assay

Cells were treated with various concentrations of IH (0-100 *μ*mol/L) for 24 h, then collected by trypsin digestion and centrifuged at 300 × g for 3 min. After washing with cold PBS, the cells were resuspended in binding buffer containing Annexin V and propidium iodide (PI; BD Biosciences, San Jose, CA, USA) and incubated for 15 min in the dark at room temperature. Analysis was performed using a FACSAria II (BD Biosciences, San Jose, CA, USA).

### 2.8. Immunocytochemistry and Terminal Deoxynucleotidyl Transferase Nick End Labeling (TUNEL)

The cells were seeded in a 6-well chamber slide and treated with 100 *μ*mol/L IH for 24 h. The steps were taken following the instructions of the TUNEL Apoptosis Detection Kit (Yisheng, Shanghai, China). Briefly, cells were fixed with 3.7% paraformaldehyde for 30 min at room temperature, washed with PBS, and incubated in 0.1% Triton X-100 for 2 min. Then, cells were incubated with TUNEL reaction complex in a humidified atmosphere at 37°C in the dark for 1 h. DAPI (1 : 5000, Invitrogen, USA) was used to label the nuclei. Fluorescence images were acquired using a fluorescence microscope.

### 2.9. Wound Healing Assay

Cells were seeded into a 6-well plate for approximately 90% confluence. When cells were attached, the confluent cell monolayers were wounded by a sterile white pipette tip. Then, the cell debris were softly washed by PBS twice and replaced with 2 mL of DMEM containing 10% FBS with different concentrations of IH (0-100 *μ*mol/L) for 24 h. The migration of cells into the cell-free space was measured by inverted microscopy; three randomly chosen fields were analyzed for each well.

### 2.10. Immunofluorescence

Tumors were fixed with 3.7% paraformaldehyde and embedded in paraffin. 5 mm thick sections were prepared. Later, tumor sections were probed with Ki67 primary antibodies (Servicebio, Wuhan, China, 1 : 200) overnight at 4°C. After washed three times, sections were incubated with secondary antibodies cy3 goat anti-rabbit IgG (Servicebio, Wuhan, China, 1 : 300) in room temperature for 1 h. DAPI was used to label the nuclei. The sections were observed under a fluorescence microscope (Carl Zeiss, Jena, Germany).

### 2.11. PFKFB4 Knockdown by Small Interfering RNA (siRNA)

B16F10 were transfected with 100 nM (final) PFKFB4 siRNA (siG180716042655; RIBO Biotechnology, Guangzhou, China) using Lipofectamine RNAiMAX reagent (Invitrogen, Carlsbad, CA, USA) according to the manufacturer's protocol. Scramble siRNA was used as a negative control (NC).

### 2.12. Tumor Model and Treatment

The protocols of our animal experiments have been approved by the Shanghai Jiao Tong University School of Medicine Animal Care and Use Committee. The effect of IH was assessed in the C57BL/6 mice (n = 6) injected with B16F10 cells. The B16F10 cells were digested by trypsin, and the cell number was adjusted at a rate of 1 × 10^5^. The mice skin was disinfected using 75% ethyl alcohol; then, 0.2 mL of cell suspension was seeded in the interior side of both left and right limbs of the mice. In this model study, IH dose was chosen 20 mg/kg per day and injected for 7 days. The mouse right limbs were injected with IH, while the left limbs were injected with same amount of saline and DMSO as the control group. The major diameter (length, L) and minor diameter (width, W) were measured every day using a caliper. Tumor volume (mm^3^) = (L × W^2^) × *π*/6. The animals were then sacrificed after 7 days.

### 2.13. Statistical Analysis

All data are expressed as the mean ± SD and were analyzed using SPSS version 18 software. Differences among groups were evaluated by one-way analysis of variance followed by Student–Newman–Keuls tests or two-way analysis of variance followed by Bonferroni post hoc tests. A *P* value of <0.05 was considered statistically significant. Statistical results are reported in the figure legends.

## 3. Results

### 3.1. Isorhamnetin Dose-Dependently Inhibits the Proliferation and Migration of B16F10 Cells

**Figure 1:**
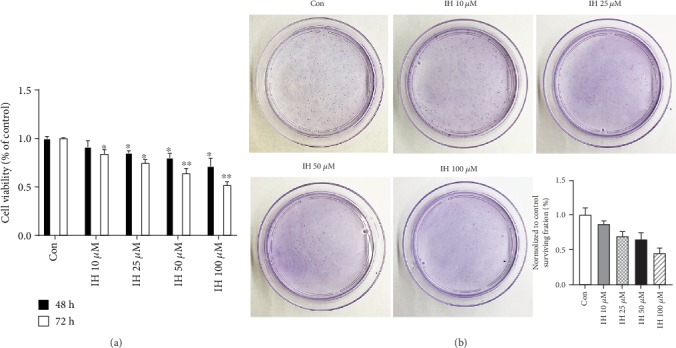
Effect of isorhamnetin on B16F10 cell proliferation. (a) Cells were treated with 0-100 *μ*mol/L IH for 48 h and 72 h. CCK-8 assay showed that cell viability was dose-dependently reduced. (b) After treated with 0-100 *μ*mol/L IH for 24 h, cells were incubated for an additional 7 days, then fixed with 3.7% paraformaldehyde, and stained with the crystal violet solution. The clonogenic assay indicated a suppressive effect of IH on B16F10 cells forming colonies. Each experiment was done at least three times. ^∗^p < 0.05 compared with control; ^∗∗^p < 0.01 compared with control.

To examine the effect of IH on B16F10 cell proliferation, we conducted CCK-8 assay and clonogenic assay. As shown in (Figure 1(a)) in CCK-8 assay, cell viability was inhibited dose-dependently after treatment with various concentrations (0-100 *μ*mol/L) of IH for 48 h and 72 h. The ability to form colonies positively correlates with cell proliferation; thus, we confirmed the result by clonogenic assay. The IH-treated cells showed decreased efficiency forming sizeable colonies in a dose-dependent manner comparing to DMSO control (Figures 1(b)).

**Figure 2:**
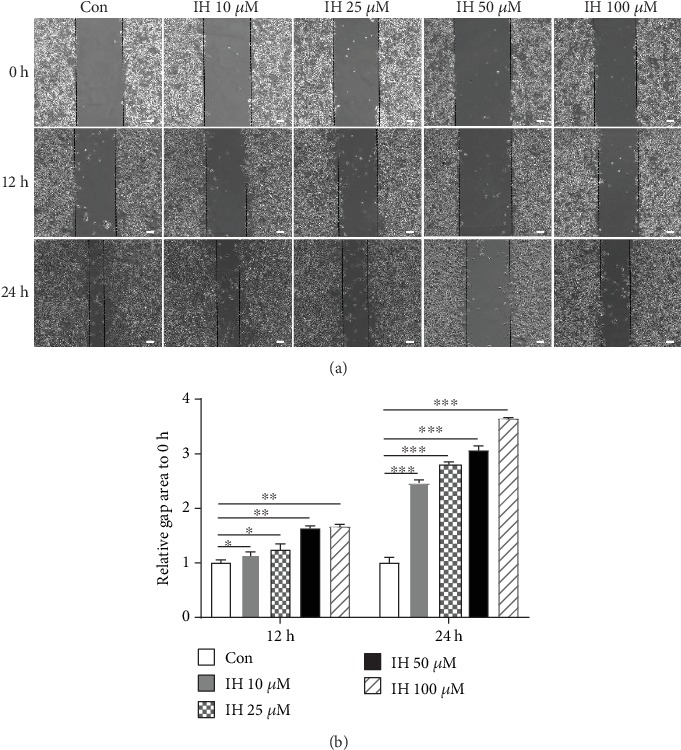
Effect of isorhamnetin on B16F10 cell migration. (a) Cells were seeded into 6-well plate and scraped with a pipette tip. After incubated with 0-100 *μ*mol/L IH for 12 h and 24 h, photographs were taken. (b) The statistical analysis was made according to the gap area compared with 0 h. IH dose-dependent and time-dependent inhibitive effects on B16F10 cell migration were observed in wound healing assay. Each experiment was done at least three times. ^∗^p < 0.05 compared with control; ^∗∗^p < 0.01 compared with control; ^∗∗∗^p < 0.001 compared with control.

Then, we investigated the inhibitive effect of IH on the migration of B16F10 cells. We analyzed the relative gap area in 12, 24 h, to 0 h gap area, with various concentrations of IH (0-100 *μ*mol/L). The wound healing assay indicated that IH concentration-dependently and time-dependently inhibited cell migration across the wounded space (Figure 2(a)). Thus, our results indicate that IH could inhibit B16F10 cell proliferation and migration in a dose-dependent manner.

### 3.2. Isorhamnetin Induces B16F10 Cell Apoptosis

**Figure 3:**
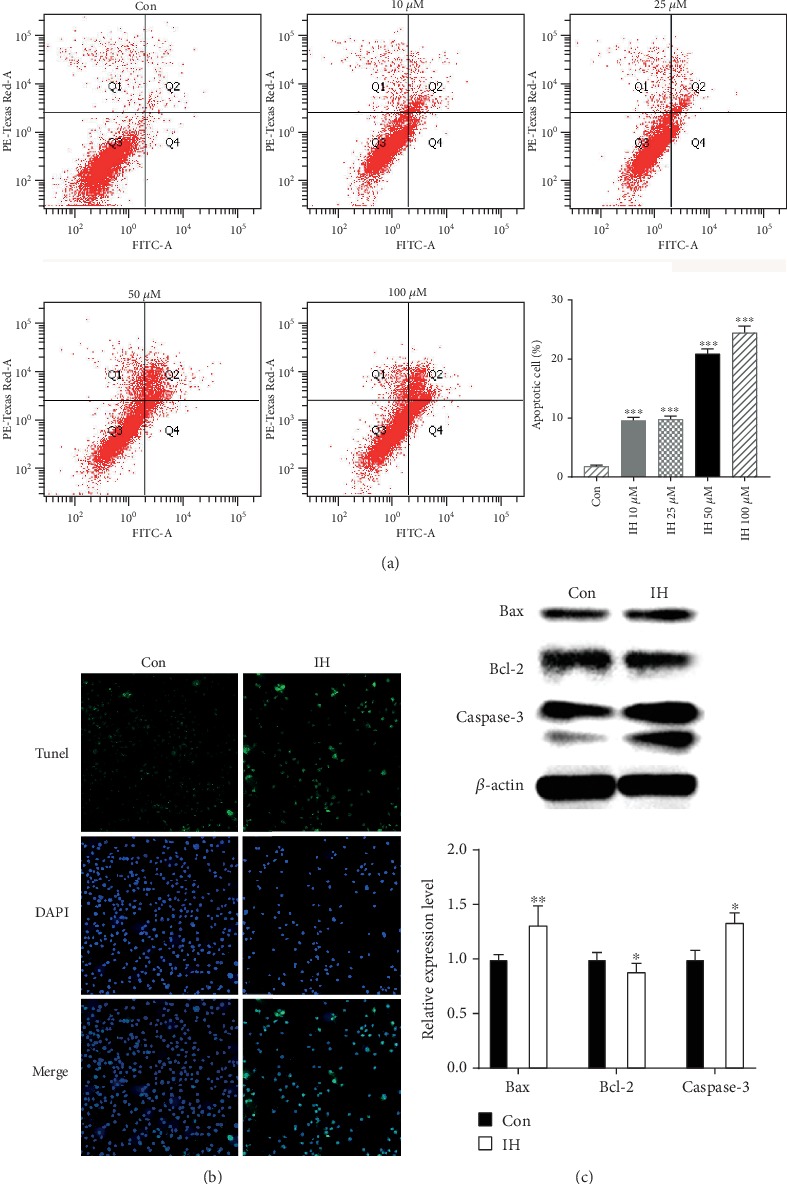
Effect of isorhamnetin on B16F10 cell apoptosis. (a) B16F10 cells were pretreated with 0-100 *μ*mol/L IH for 24 h and then resuspended in binding buffer containing Annexin V and PI. Cell suspension was analyzed by FACSAria II, which indicated cell apoptosis induced by IH dose-dependently. (b) Representative images of DAPI staining and Tunel assay conducted to analyze B16F10 cell apoptosis. (200x) (c) The expression of Bax, Bcl-2, and Caspase-3 were analyzed by Western blotting with specific antibodies. The levels of Bax and Caspase-3 were upregulated, and Bcl-2 was downregulated after treatment with 100 *μ*mol/L IH. The anti-*β*-actin antibody was used to check the proper protein loading. Each experiment was repeated at least three times. ^∗^p < 0.05 compared with control; ^∗∗^p < 0.01 compared with control; ^∗∗∗^p < 0.001 compared with control.

To determine whether the reduction of B16F10 cell viability induced by IH was correlated with cell apoptosis or necrosis, we conducted Annexin V/PI double staining and flow cytometry analysis. The result showed that IH induced cell apoptosis in a dose-dependent way (1.8% DMSO control versus 24.2% 100 *μ*mol/L IH-treated group, Figure 3(a)). The result was further confirmed by TUNEL staining assay (Figure 3(b)). The number of apoptotic cells was significantly increased after incubation of IH (100 *μ*mol/L) compared with DMSO control.

In addition, the intrinsic apoptotic pathway is basically regulated by proteins that belongs to the Bax family and the Caspase family. Hence, the alterations of proapoptotic proteins, Bax and Caspase-3, and anti-apoptotic protein, Bcl-2, could affect the apoptosis induction. We conducted Western blot of B16F10 cells treated with 100 *μ*mol/L IH for 24 h. The expression of Bax and Caspase-3 was upregulated, whereas the expression of Bcl-2 was downregulated (Figure 3(c)). Thus, these data verified the ability of IH-inducing B16F10 cell apoptosis.

### 3.3. Isorhamnetin Inhibits PI3K/Akt and NF-*κ*B Pathways

**Figure 4:**
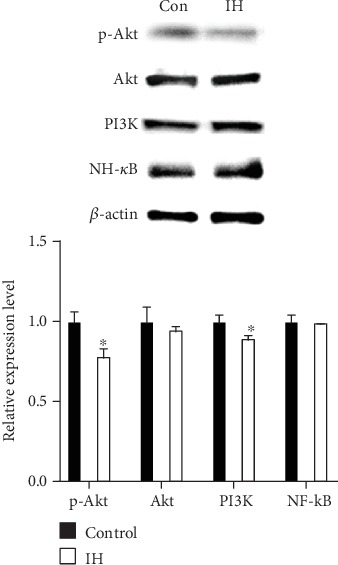
Effect of isorhamnetin on the PI3K/Akt and NF-*κ*B pathways in B16F10 cells. Cells were pretreated with 100 *μ*mol/L IH for 24 h; then, the cell extracts were blotted with specific antibodies. The phosphorylated protein level of p-Akt was detected. The level of p-Akt and total PI3K was downregulated after IH treatment, and the total level of Akt and NF-*κ*B showed no difference. The anti-*β*-actin antibody was used to check the proper protein loading. Each experiment was repeated at least three times. ^∗^p < 0.05 compared with control.

The underlying molecular mechanism of IH on B16F10 cell was further investigated. Because the PI3K/Akt pathway plays a pivotal role in cell survival and proliferation, we examined its activity by Western blot under IH treatment. The expression of total PI3K and phosphorylated Akt were markedly decreased by IH (100 *μ*mol/L). However, the expression level of total Akt was not affected (Figure 4). These results indicated that IH could inhibit the activation of the PI3K/Akt pathway.

**Figure 5:**
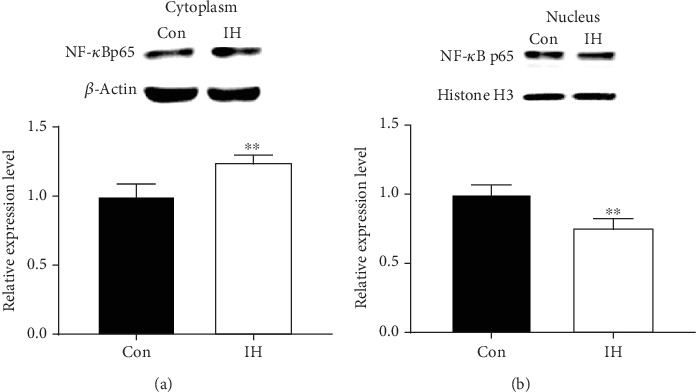
Effect of isorhamnetin on NF-*κ*B in B16F10 cells. Cytosolic extracts and nuclear extracts were prepared after incubated with 100 *μ*mol/L IH for 24 h. (a, b) The translocation of NF-*κ*B p65 was detected by Western blotting. All proteins were determined with specific antibodies. The suppressive effect of IH was confirmed by the upregulation of cytosolic NF-*κ*B and downregulation of nuclear NF-*κ*B. The anti-*β*-actin antibody and anti-Histone H3 were used to check the proper protein loading. Each experiment was repeated at least three times. ^∗^p < 0.05 compared with control.

PI3K/Akt activation could phosphorylate I*κ*B, which is an endogenous inhibitor combined with NF-*κ*B p65. When phosphorylated, NF-*κ*B p65 releases from I*κ*B and translocates to cell nucleus. Our Western blot result indicated that, after 24 h incubation with 100 *μ*mol/L IH, the level of total NF-*κ*B stay unaffected (Figure 4). Moreover, the level of cytosolic NF-*κ*B was increased, whereas the nuclear NF-*κ*B was decreased (Figures 5(a) and 5(b)). These results showed evidence that the NF-*κ*B pathway was inhibited by IH.

### 3.4. PFKFB4 Is Involved in Isorhamnetin-Induced PI3K/Akt Pathway Inhibition

**Figure 6:**
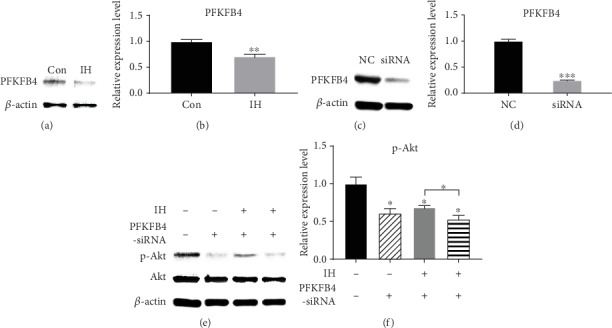
Effect of PFKFB4 on isorhamnetin-induced PI3K/Akt pathway inhibition. (a, b) When treated with IH (100 *μ*mol/L), the PFKFB4 expression level was significantly decreased. (c, d) PFKFB4 expression was downregulated after treatment with specific siRNA. (e, f) Effect of PFKFB4 on the PI3K/Akt pathway in IH-treated B16F10 cells. Each experiment was repeated at least three times. ^∗^p < 0.05 compared with control; ^∗∗^p < 0.01 compared with control; ^∗∗∗^p < 0.001 compared with control.

PFKFB4 has been reported to have increased expression in various malignant cancer. [14] We discovered that when treated with IH (100 *μ*mol/L), the PFKFB4 expression level was significantly decreased (Figures 6(a) and 6(b)). The knockdown experiment of PFKFB4 was conducted to verify whether IH could inhibit the expression of PFKFB4 to suppress the phosphorylation of Akt (Figures 6(c) and 6(d)). The results showed that the inhibiting effect of IH on p-Akt was enhanced by PFKFB4-specific siRNA (Figures 6(e) and 6(f)), suggesting that PFKFB4 depletion affected Akt phosphorylation under IH treatment. Thus, we concluded that PFKFB4 played a role in IH-induced PI3K/Akt inhibition. Based on former results, we further hypothesize that IH might suppress the PI3K/Akt pathway in B16F10 cells through targeting PFKFB4.

### 3.5. Isorhamnetin Suppresses B16F10 Cell Proliferation In Vivo

**Figure 7:**
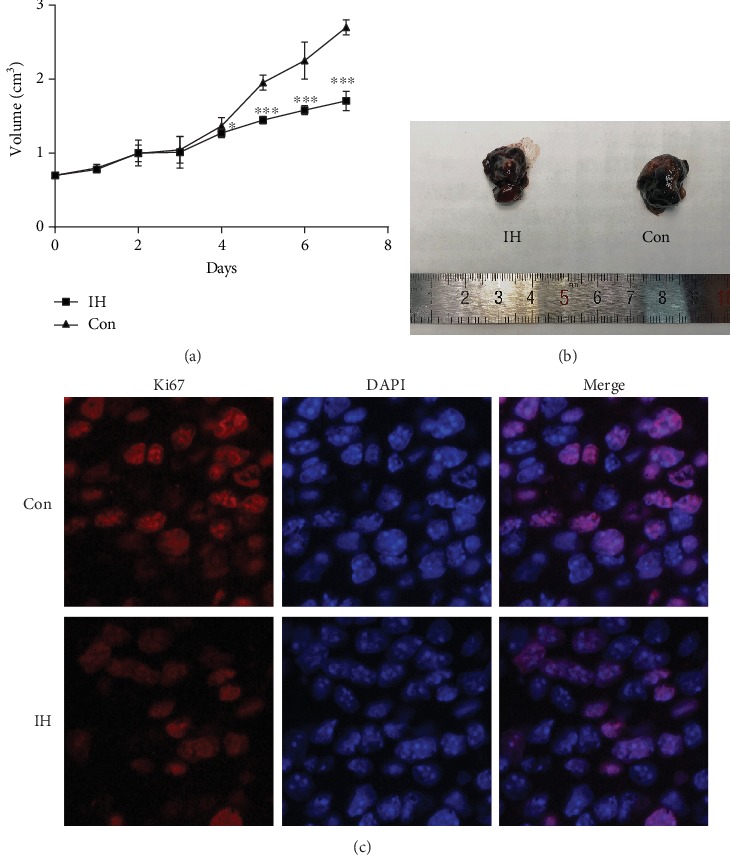
Effect of isorhamnetin of B16F10 cell proliferation in vivo. B16F10 cells were injected into C57BL/6 mice subcutaneously, then treated with 20 mg/kg IH or DMSO control every day for 7 days (n = 6). (a) The survival curve was based on tumor volumes. The tumor in the IH group developed slower than control. (b) Representative image of the macroscope at the 7^th^ day. (c) Ki67 fluorescence was taken to examine melanoma proliferation. Each experiment was repeated at least three times. ^∗^p < 0.05 compared with control; ^∗∗^p < 0.01 compared with control; ^∗∗∗^p < 0.001 compared with control.

To further confirm the inhibitory effect of IH on B16F10 cells in vivo, C57BL/6 mice were adopted for B16F10 cell injection. In this model study, IH dose was given 20 mg/kg per day. The tumor sizes were measured and recorded every day. The survival curve was made based on tumor sizes (Figure 7(a)). After 7 days, a remarkable difference in the tumor size was observed (Figure 7(b)). The volume of the IH group was 1.58 cm^3^, and the DMSO control was 2.25 cm^3^. Immunofluorescence detected that Ki67 was decreased in the IH-treated group, which indicated the inhibitive effect on proliferation of IH (Figure 7(c)). However, the TUNEL-positive staining assay showed no significant difference in the apoptotic cell number. These results suggested that IH could inhibit B16F10 cell proliferation in vivo.

## 4. Discussion

In this study, we demonstrated for the first time that as a natural compound IH could significantly induce cell apoptosis in melanoma. Our results indicated that IH attenuated B16F10 melanoma cell proliferation and migration and promoted cell apoptosis in a dose-dependent manner. The underlying mechanisms in this process involve the downregulation of thePI3k/Akt and NF-*κ*B pathways.

Previously, several studies have focused on the effect of IH on the inhibition of cell proliferation as well as induction of apoptosis on various types of tumors, including nonmelanoma skin cancer (NMSC) [15] and bladder cancer [16]. In light of these findings, we investigated the antitumor activity of IH in B16F10 melanoma cell lines in vitro and in vivo. The inhibitive effect of proliferation was determined by CCK-8 assay and clonogenic assay, which showed that IH could promote cell death in a dose-dependent manner. The cell growth inhibition could be seen at dosage as low as 10 *μ*mol/L, and the maximal dosage reached 100 *μ*mol/L. To further confirm these visual changes, the flow cytometry was performed with Annexin V/PI staining, and TUNEL assay was also taken. The results also indicated that IH could induce B16F10 cell apoptosis. Besides, according to wound healing assay, IH could effectively inhibit cell migration in a concentration-dependent manner. Thus, our study demonstrated that IH suppressed the proliferation and migration of B16F10 cell, which may act as a potential drug in melanoma. In accordance with in vitro findings, the in vivo experiments suggested suppressive proliferation of IH treated tumors by measuring tumor sizes. And the macroscope outcome was confirmed when conducting Ki67 immunofluorescence.

The intrinsic apoptosis pathway is mainly regulated by proteins of the Bax family and the Caspase family. After treated with IH, the levels of Bax and Caspase-3 were significantly increased and the level of Bcl-2 was decreased; thereby, the ratio of Bax/Bcl-2 was largely increased. As Bax and Caspase-3 are proapoptotic proteins while Bcl-2 is an antiapoptotic protein, this result further verified that IH could induce apoptosis in B16F10 cell. Combining with former results, we can reasonably infer that IH could be a potent trigger in cancer cell apoptosis and thus become a prophylactic agent in tumor.

PI3K has been acknowledged to involve in numbers of fundamental cellular activities, such as proliferation, apoptosis, and motility. [17] Not only in normal cells, PI3K/Akt pathway is upregulated in tumorgenesis and considered a key regulator in cancer cells [18]. Moreover, some kinds of flavonoid, such as quercetin and its analogs, are inhibitors of PI3K. [19, 20] There are also evidences suggesting that IH induces apoptosis via the PI3K and MEK pathways. [11, 15] In view of former studies, we investigated the effect of IH on PI3K, Akt, and its phosphorylated form in B16F10 cell by Western blot. The results showed that IH suppressed PI3K and phospho-Akt expression, while the total expression of Akt stayed unaffected. As Akt is a key downstream factor of PI3K, it might reflect the whole status of the PI3K/Akt pathway. These were consistent with a previous study which proved the cytostatic effect of IH on breast cancer through the Akt and mitogen-activated protein kinase kinase (MAPK) signaling pathways. [12] Hence, we identified that IH could downregulate the PI3K/Akt pathway.

Moreover, NF-*κ*B activation has been observed to play an important role in proliferation and metastasis in many cancer cells, including melanoma. [21–23] It has also been reported that activated Akt could activate NF-*κ*B by phosphorylating I*κ*B in various cancer cells, so the suppressive effect of IH on the PI3K/Akt pathway may contribute to the inhibitory effect of NF-*κ*B. [24, 25] Normally, NF-*κ*B exists in the cytoplasm in a form of inactive heterodimer containing p50 and p65 subunits which bound to an inhibitory monomer I*κ*B. When stimulators activate I*κ*B kinase (IKK), I*κ*B is phosphorylated and degraded; thereby, NF-*κ*B releases from I*κ*B complex, then translocates to the nucleus, and enhances the expression of a variety of genes. We conducted the Western blot analysis of total NF-*κ*B, NF-*κ*B in the cytoplasm and the nucleus. The results showed that the level of total NF-*κ*B stayed unaffected, while nuclear NF-*κ*B was decreased and cytoplasmic NF-*κ*B was increased. Thus, our study indicated that IH inhibits the translocation of NF-*κ*B and the activation of the NF-*κ*B pathway. The suppressive effect of IH on the NF-*κ*B pathway has been investigated in different cancer cell lines; most of which were in accordance with our results. [12, 26, 27] Interestingly, a study of esophageal squamous carcinoma reported that the NF-*κ*B pathway showed activation in the first 72 h exposure of IH but became inhibited later. Explanation of this phenomenon was that cellular stress response (involving NF-*κ*B activation) inhibited cell apoptosis in the early exposure [10]. However, cell apoptosis dominated later, leading to NF-*κ*B inhibition. [10] The underlying mechanism of this variation still needed further study, yet the difference between cell lines, the duration of IH treatment, and cellular stress response should be taken into account in future exploration.

In addition, PFKFB4 is an enzyme of the PFKFB family, which is the rate-limiting enzyme in glycolysis. [28, 29] PFKFB4 was reported to play a vital role in tumor cell growth, proliferation, and survival. [30] Comparing with normal tissues, increased expression of PFKFB4 was demonstrated in gastric cancer, pancreatic cancer, and glioblastoma, and more robust expression was shown in breast and lung cancer. [14, 31] Therefore, we were curious to investigate whether IH affect PFKFB4 in melanoma cell line. We hypothesized that IH could affect PFKFB4, thereby influence the activation of the PI3K/Akt pathway. Our knockdown experiment of PFKFB4 also confirmed that PFKFB4 selectively inhibits the PI3K/Akt pathway in the presence of IH, which proved that PFKFB4 plays a role in the transduction effect of IH in the PI3K/Akt pathway. The interaction between PFKFB4 and Akt signaling was also reported recently. [32, 33] Pegoraro et al. observed that PFKFB4 depletion led to reduction of Akt phosphorylation, but no direct relationship was established in the research. Our results are preliminary in PFKFB4 molecular mechanism; further studies are needed to be done to detect the role of PFKFB4 in the PI3K/Akt pathway.

The relationship between PFKFB4 and the NF-*κ*B pathway has not been investigated so far. However, numbers of researches have observed a reactive oxygen species (ROS) increase after PFKFB4 depletion. [30, 34, 35] As NF-*κ*B is a major transcriptional factor of inflammation, it could induce amounts of proinflammatory gene expression, such as ROS, COX-2, and TNF-*α*. [36] Thus, we proposed that PFKFB4 may be required in IH effect through the ROS-reliant NF-*κ*B pathway. Investigation of PFKFB4 role in NF-*κ*B-related inflammation process is essential, which points out the direction for further research.

In conclusion, our study demonstrated for the first time that IH, a natural flavonoid compound, could effectively attenuate the proliferation and migration of B16F10 melanoma cell line. We also revealed that the IH antitumor effect was through inducing cell apoptosis and confirmed that by the alter of Bax, Bcl-2, Caspase-3 expression, and TUNEL staining. Furthermore, studying the underlying molecular mechanism, we suggested that IH inhibited the PI3K/Akt pathway and the NF-*κ*B pathway in B16F10 cells, and the inhibitory effect of IH was related to PFKFB4. The in vivo experiments verified the suppressive proliferation of melanoma tumors under IH treatment by tumor sizes and Ki67 immunofluorescence. Taken together, this compound could serve as a promising candidate in melanoma therapy. Nevertheless, this study merely focused on IH proapoptosis effect; further in-depth research in its molecular mechanism and clinical application are still needed.

## Data Availability

The materials and relevant data will be freely available to any scientist for noncommercial purposes from the corresponding author.
